# Online event-triggered switching load frequency control of islanded microgrids through machine learning

**DOI:** 10.1007/s44291-026-00218-7

**Published:** 2026-05-05

**Authors:** Osita U. Omeje, Luciano Olukanni, Daniel E. Okojie, Candidus U. Eya, Abiola B. Aina

**Affiliations:** 1 Department of Electrical and Electronics Engineering, University of Lagos, Lagos, Nigeria; 2 Department of Electrical and Electronic Engineering, Pan-Atlantic University, Km 52 Lekki-Epe Expressway, Ibeju-Lekki, Lagos, Nigeria; 3 Department of Electrical Engineering, University of Nigeria, Nsukka, Enugu state Nigeria

**Keywords:** Distributed energy resources, Load frequency control, Machine learning, Microgrids, Neural PI control, Online event triggered control, Renewable energy resources

## Abstract

This paper presents the design and development of an adaptive online event-triggered load frequency control (LFC) framework for islanded microgrids using machine learning techniques. The proposed approach addresses the stability challenges and performance limitations of conventional LFC strategies arising from the dynamic and stochastic characteristics of modern microgrids with high renewable penetration. A Neural-PI controller, pre-trained to capture complex system dynamics, is employed to enable intelligent event-triggered switching and real-time adjustment of control gains, thereby enhancing frequency regulation and overall system stability. The framework is implemented in Python using a Jupyter Notebook environment and evaluated on the IEEE 14-bus test system, where results demonstrate its ability to dynamically adapt event thresholds under varying operating conditions, leading to improved stability and control efficiency. Accordingly, the Neural-PI controller markedly outperforms the conventional PID controller, achieving a 58% reduction in peak frequency deviations, a 33% improvement in settling time, and a 95% reduction in control actions through event-triggered switching, with particularly strong performance during periods of renewable intermittency. Its adaptive capability enabled 2.5 times more effective inertia mode changes, reduced oscillations and overshoot, and contributed to a 19% increase in renewable hosting capacity alongside a 37% reduction in load-frequency control operational costs, making it especially suitable for islanded and low-inertia grids.

## Introduction

### Motivation

Economic, political, and technological drivers have accelerated the global transition toward clean and sustainable energy systems, leading to significant transformations in the modern energy landscape [1]. Within this context, microgrids (MGs) have emerged as advanced localized power systems capable of operating either in grid-connected or islanded modes, thereby offering enhanced flexibility, reliability, and sustainability [2]. Their ability to continue operation during grid outages makes them particularly attractive for critical infrastructures such as hospitals, military installations, and emergency response facilities, while also supporting the broader transition toward decentralized energy systems [3, 4].

In islanded operation, a microgrid functions as a self-contained power system that relies exclusively on local distributed generation (DG) sources, including solar photovoltaic systems, wind turbines, and small-scale generators [5]. Although this configuration reduces transmission losses and improves overall efficiency, it introduces significant operational challenges due to the inherent variability and intermittency of renewable energy sources. Maintaining energy self-sufficiency, therefore, requires continuous dynamic load balancing, as even small mismatches between generation and demand can cause unacceptable deviations in system frequency and voltage [3]. To mitigate these effects, energy storage systems (ESS) such as lithium-ion batteries and flywheels are widely deployed to absorb excess energy and rapidly compensate for short-term fluctuations, thereby enhancing microgrid reliability, especially at high renewable penetration levels [6].

**Fig. 1:**
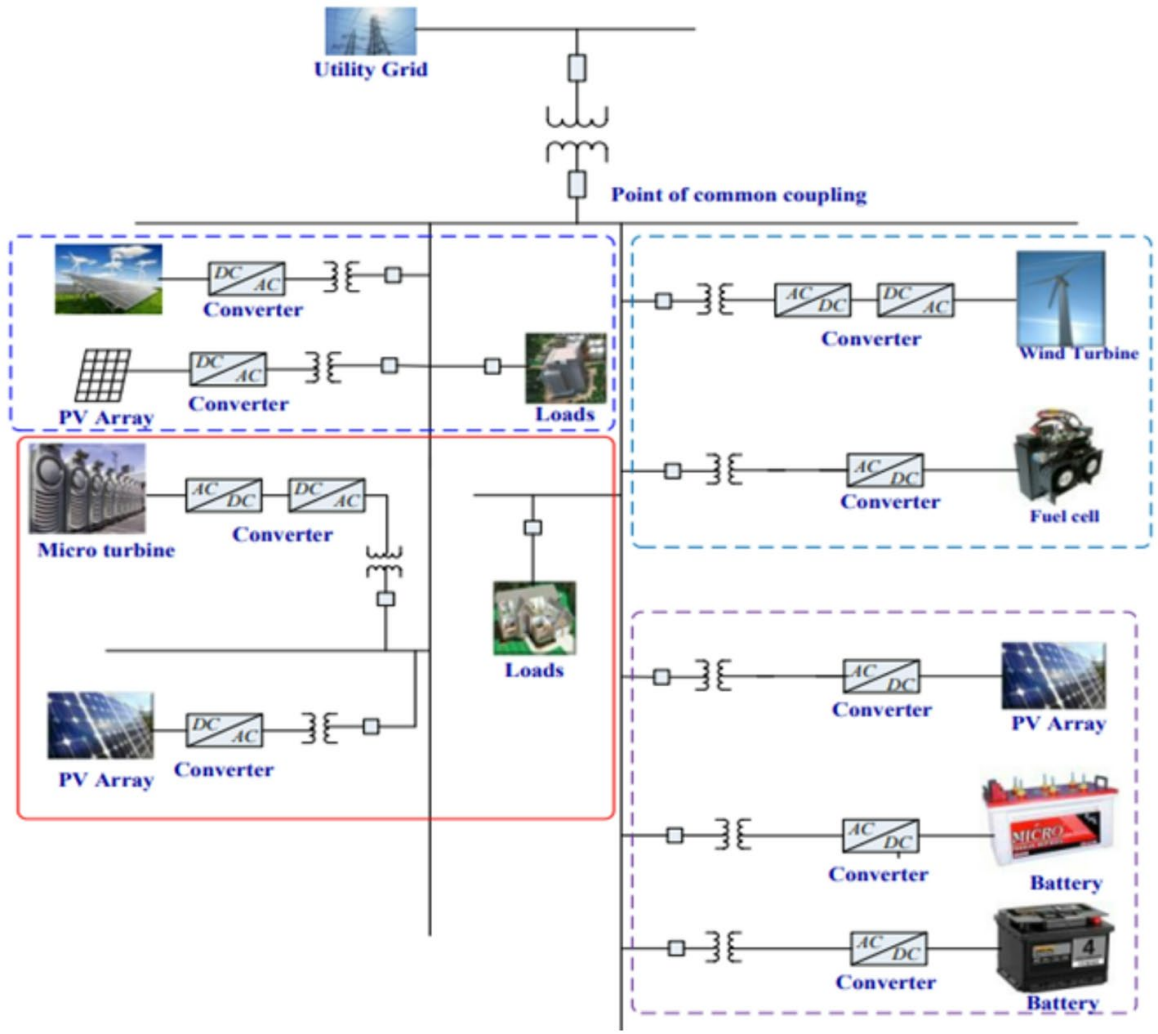
A typical microgrid control system

Advanced monitoring, coordination, and control infrastructures further support stable microgrid operation by enabling real-time supervision of generation, storage, and load profiles [7]. These systems (with a typical example shown in Fig. 1) increasingly integrate modern technologies such as the Internet of Things, machine learning, and cloud computing to optimise energy conversion, coordinate distributed resources, and ensure secure, autonomous operation. Effective frequency and voltage regulation remain central to microgrid stability, as sudden load changes or generation losses require immediate corrective actions to protect equipment and maintain power quality [8, 9]. The resilience of islanded microgrids is further strengthened by features such as black-start capability, which allows autonomous recovery following major disturbances.

Despite these advantages, islanded microgrids face notable challenges, including limited generation capacity, stringent real-time balancing requirements, and the variability of renewable energy sources such as solar and wind [10]. Traditional load frequency control (LFC) methods, which depend on spinning reserves and continuous control actions, are often unsuitable for islanded microgrids due to resource constraints, sparse communication infrastructure, and reduced system inertia [11–13]. As a result, event-triggered control (ETC) has gained attention as a resource-efficient alternative, executing control actions only when predefined conditions are met. However, conventional ETC schemes rely on fixed thresholds, making them inflexible and suboptimal under rapidly changing operating conditions. This limitation has motivated the development of adaptive event-triggered control (AETC) strategies, which dynamically adjust control thresholds in real time to improve frequency stability while minimizing communication and control effort [14].

To implement these control strategies effectively, microgrids adopt various control architectures, including centralized, decentralized, distributed, optimised, and hierarchical frameworks, each offering distinct trade-offs in terms of scalability, robustness, coordination, and complexity [15–19]. Advanced control techniques such as model predictive control (MPC) and fuzzy logic control further enhance adaptability and robustness by addressing system constraints, nonlinearities, and uncertainties, albeit at the cost of increased computational and design complexity [20]. Collectively, these developments underscore the growing importance of intelligent, adaptive control frameworks in ensuring the stable, efficient, and resilient operation of islanded microgrids with high renewable energy penetration.

Adaptive and intelligent control strategies play a critical role in ensuring stable and efficient microgrid operation under varying and uncertain conditions. Adaptive control dynamically adjusts control parameters in real time to accommodate changing system dynamics, improving performance and robustness, although its practical implementation can be complex and may introduce instability if not properly designed and tuned [21]. Event-triggered control reduces communication and computational burden by initiating control actions only when predefined events or conditions occur, enabling efficient resource utilization; however, its effectiveness depends heavily on accurate event detection and appropriate threshold selection to avoid missed or delayed responses [22].

In parallel, demand response strategies contribute to microgrid stability by actively modifying consumer demand in response to grid conditions, thereby reducing peak loads and enhancing overall system balance, though their success relies on user participation and robust communication infrastructure [23]. The energy management system (EMS) serves as a supervisory layer that coordinates generation, storage, and load management to optimise energy use, facilitate renewable energy integration, and improve system reliability and resilience, albeit at the cost of increased implementation complexity and reliance on accurate real-time data [24].

A comparative summary of these microgrid control and management approaches is provided in Table 1.

**Table 1:** Summary of microgrid system control architecture

Control Strategy	Description	Advantages	Disadvantages
Centralized Control	A single controller manages the entire Microgrid	Simple implementation and management	Vulnerable to single points of failure
Decentralized Control Strategy	Individual components operate autonomously based on local measurements	Increased reliability and resilience	Coordination challenges
Distributed Control	Local controllers collaborate for optimal system performance	Improved coordination and resource utilization	Complexity in communication
Hierarchical Control	Control tasks are divided into multiple levels (primary, secondary, tertiary)	Clear delineation of responsibilities	Complex design and potential delays
Model Predictive Control (MPC)	Uses a model to predict future states and optimise control actions	Handles constraints, provides optimal actions	Computationally intensive
Fuzzy Logic Control	Employs fuzzy rules to manage uncertainties	Handles non-linearities effectively	Performance depends on rule design
Adaptive Control	Adjusts parameters in real-time based on changing dynamics	Responsive to varying conditions	Complexity in tuning and potential instability
Event-Triggered Control	Executes actions based on specific events rather than time intervals	Reduces load and efficient resource use	Risk of missing events
Demand Response Control	Adjusts consumer demand in response to grid conditions	Reduces peak demand and enhances stability	Requires consumer engagement and infrastructure
Energy Management Systems (EMS)	Oversees the operation and control of the microgrid	Optimises generation and consumption	Can be complex and costly to implement

### Evolution of microgrid load frequency control strategies

Researchers have extensively investigated LFC architectures to address the operational challenges of microgrids (MGs), particularly those arising from the intermittent and stochastic nature of renewable energy sources (RES). The effectiveness and reliability of an MG largely depend on the choice of LFC strategy, which must ensure frequency stability under rapidly varying generation and load conditions. Existing approaches, however, often exhibit inherent limitations, motivating the continuous search for more robust and efficient control solutions.

Broadly, MG LFC strategies can be classified into traditional, advanced, and hybrid methods. Traditional LFC techniques such as proportional–integral–derivative (PID), proportional–integral (PI), droop control, automatic generation control (AGC), tie-line bias control, load shedding, peak shaving, and spinning reserve-based approaches are simple and well established but generally lack the adaptability required for high RES penetration. In contrast, advanced LFC methods, including model predictive control (MPC), adaptive control, artificial intelligence–based techniques (e.g., neural networks, fuzzy logic, and machine learning), distributed and robust control, sliding mode control, optimal control, energy storage–based control, virtual synchronous generator (VSG) control, and event-triggered control, offer improved robustness and dynamic performance at the expense of increased complexity.

To overcome the limitations of individual approaches, hybrid LFC strategies combine traditional and advanced techniques, or multiple advanced methods, to exploit their complementary strengths. Examples include neural network sliding mode control for nonlinear MG dynamics, fuzzy logic MPC for improved performance under uncertainty, fuzzy-PID for enhanced adaptability with implementation simplicity, game-theoretic distributed control for coordinated decision-making, and AI-assisted robust control for uncertainty mitigation. A comparative summary of these LFC strategies is presented in Table 2.

**Table 2:** Summary of microgrid LFC strategies

Control strategy	Description	Advantages	Disadvantages
Traditional or conventional control method	A single controller manages the entire Microgrid	Simple, cost effective and reliable mostly for conventional power system	Vulnerable to single points of failure. Inadequate for complex, dynamic, modern grids
Advanced Control method	Individual components operate autonomously based on local measurements	Ensure enhanced performance, adaptability, frequency stability in modern complex MG	High implementation cost, complexity and coordination challenges
Hybrid control method	Local controllers collaborate for optimal system performance	Improved coordination, resource utilization and robustness with frequency stability	Increased cost, complexity in tuning and implementation

Extensive research on traditional LFC methods for microgrids (MGs) is reported in [21], [23–36], with a dominant focus on droop control, PI, and PID controllers. These studies highlight the simplicity, low cost, and industrial relevance of classical controllers, while also identifying inherent challenges related to tuning sensitivity, limited adaptability, and performance degradation under nonlinearities and high renewable energy source (RES) penetration. For instance, a modified droop control strategy for islanded MGs using parallel inverters was proposed in [33], mimicking synchronous generator behaviour to achieve improved voltage and frequency regulation. Although its performance was comparable to conventional droop control, challenges such as circulating currents, power-sharing accuracy, communication latency, and robustness persisted.

Observer-based and optimal control approaches have also been explored. In [31], a PI-observer-based state feedback scheme demonstrated improved disturbance rejection and robustness compared to Luenberger observers, but its reliance on accurate system modelling limited applicability under large uncertainties. Similarly, PID-based designs for networked control systems were examined in [36], showing feasibility when communication constraints are considered during tuning, albeit with limitations in scalability, adaptability, and nonlinear system handling. Classical automatic generation control (AGC) enhancements, including time error correction [37] and advanced PID variants such as PIDD [38, 39], improved closed-loop performance but inadequately addressed parameter uncertainty, fault tolerance, and adaptability.

Optimal and suboptimal control strategies including linear optimal control, minimum settling time theory, and linear quadratic regulator (LQR)-based LFC were investigated in [32, 41, 42]. These methods demonstrated enhanced frequency regulation and tie-line power control through systematic optimisation and pole placement. However, their practicality was constrained by high computational demands, dependence on full-state availability, and sensitivity to modelling inaccuracies. Recent efforts to improve classical controllers via optimisation techniques, such as gradient-based optimisers for PID tuning [36], achieved superior dynamic performance compared to conventional PID designs, yet retained limited robustness under large operating variations.

Overall, the literature confirms that traditional LFC methods remain foundational for frequency regulation and provide valuable insights into control design and stability analysis. Nevertheless, their limitations slow dynamic response, poor adaptability to RES intermittency, inability to handle nonlinearities, fixed-parameter operation, scalability issues, and dependence on reliable communication restrict their effectiveness in modern MGs. These shortcomings have driven the evolution toward advanced and hybrid LFC strategies.

Accordingly, advanced LFC techniques reported in [37–57] leverage adaptive control, artificial intelligence, robust control, optimisation algorithms, and event-triggered schemes to address uncertainty, variability, and low inertia in MGs. Adaptive fuzzy MPC approaches [55] demonstrated superior frequency regulation compared to conventional MPC, albeit at the cost of increased computational complexity. Virtual synchronous generator (VSG) designs combined with robust H∞ control [56] improved stability but introduced conservatism and implementation challenges. Event-triggered control (ETC) schemes [57, 58] effectively reduced communication and computation overhead while maintaining stability, though their sensitivity to RES uncertainty and spinning reserve constraints remained problematic.

Hybrid approaches integrating adaptive, robust, intelligent, and event-triggered mechanisms [57, 59] further enhanced performance in islanded MGs with limited inertia and spinning reserves. Notably, the bounded adaptive event-triggered LFC mechanism (BAETM) proposed in [60] addressed spinning reserve constraints by bounding triggering thresholds to improve monitoring sensitivity and reduce unnecessary communication. Despite its merits, BAETM suffers from design complexity, tuning difficulty, computational burden, and sensitivity to modelling errors, limited scalability, and potential instability if poorly configured.

In light of these identified gaps, there remains a clear need for refined BAETM-based LFC strategies that improve adaptability, robustness, and efficiency while reducing implementation complexity and computational overhead. Addressing these challenges as indicated in Table 3, is essential for achieving reliable frequency regulation in RES-dominated, low-inertia islanded microgrids.

**Table 3:** Summary of BAETM drawbacks

Drawbacks	Descriptions
Complexity in Design	Challenges in setting appropriate error bounds; requires accurate system modelling
Potential for Instability	Risks of instability if bounds are poorly chosen; can lead to chattering in control inputs
Sensitivity to Disturbances	May not handle transient disturbances well; performance sensitive to parameter variations
Increased Computational Load	Calculation of error and bounds may add computational burden; potential delays in control actions
Implementation Challenges	Difficulties in synchronizing bounds in distributed systems; possible communication overhead
Limited Applicability	Not suitable for systems requiring continuous updates; may not perform well in rapidly changing conditions
Difficulties in Tuning	Requires parameter tuning which can be challenging; balancing responsiveness and efficiency is complex

In summary, although the BAETM offers a promising framework for reducing communication and computational burden while maintaining control performance, its practical effectiveness depends strongly on careful design and tuning. Challenges related to bound selection, robustness to uncertainties, computational overhead, and implementation complexity can limit performance if not properly addressed. Consequently, improving BAETM requires strategies that preserve its event-triggering advantages while enhancing adaptability, robustness, and smooth control action.

Key improvement directions include adaptive bound adjustment, where upper and lower thresholds are dynamically updated based on system performance, disturbance levels, or operating conditions. Learning-based techniques can further enhance this process by identifying near-optimal bounds from historical and real-time data. To improve resilience against modelling uncertainties and disturbances, robust control integration, supported by rigorous stability analysis, can be incorporated within the bounded framework.

Enhancements to the event-triggering strategy itself such as multi-level triggering and hierarchical control architectures enable more nuanced responses to varying error magnitudes and critical operating conditions. The integration of state observers and adaptive estimators improves state awareness and reduces the impact of noise and unmeasured disturbances, leading to more informed triggering decisions. In addition, feedback-driven adaptation allows periodic assessment of control performance and continuous refinement of bounds and control parameters.

Data-driven and learning-enabled approaches further strengthen BAETM performance. Simulation-based validation, real-time data analysis, reinforcement learning, and hybridization with predictive schemes such as model predictive control (MPC) support proactive and context-aware control actions. In distributed settings, enhanced communication and cooperative control mechanisms help synchronize triggering decisions while minimizing network overhead.

Finally, ensuring smooth control action through signal filtering and rate limiting mitigates chattering near triggering thresholds, improving actuator longevity and system stability. The use of well-defined performance metrics and benchmarking against alternative control strategies provides objective evaluation and supports continuous improvement.

Overall, by integrating adaptive, robust, learning-based, and hierarchical enhancements, BAETM can be significantly refined to achieve higher efficiency, robustness, and scalability in practical microgrid load frequency control applications as summarized in Table 4.

**Table 4:** Summary of Improvement strategies for BAETM

Improvement strategy	Description
Adaptive Bound Adjustment	Dynamically adjust bounds based on performance or disturbances
Robust Control Techniques	Incorporate robust control methods for uncertainty management and stability analysis
Event-Triggered Updating	Implement multi-level triggers and hierarchical control structures for nuanced responses
Integrated Observer Design	Use state observers for better predictions and adaptive parameters
Feedback Mechanisms	Introduce feedback loops to assess and adjust bounds and strategies periodically
Data-Driven Approaches	Utilize simulations and real-time data analysis for optimal tuning of control strategies
Enhanced Communication Protocols	Optimise communication in distributed systems for better coordination
Incorporating Learning Algorithms	Use reinforcement learning or MPC for adaptive control policies
Ensuring Smooth Control Actions	Apply smoothing techniques and rate limiting to reduce chattering effects
Performance Evaluation Metrics	Establish clear metrics for evaluating BETC performance and benchmarking against other strategies

By adopting the improvement strategies outlined in Table 4, the effectiveness of the bounded adaptive event-triggered mechanism (BAETM) can be substantially enhanced in terms of stability, responsiveness, and overall control efficiency. In this work, adaptive bound adjustment is proposed as the primary enhancement strategy to address the identified limitations of BAETM, with particular emphasis on a learning-based approach that leverages machine learning to infer optimal triggering bounds from historical data and observed system behaviour. Specifically, pre-trained Neural–PI controllers are employed to realize adaptive bound adjustment in real time, enabling the control system to better accommodate dynamic operating conditions and uncertainties.

Although prior studies have explored neural controllers, Neural–PI structures, and machine learning techniques for power system stability and control, as reported in [61–76], several gaps remain. For instance, the approach in [69] successfully bridges neural network-based control and formal stability guarantees by employing equilibrium-independent passivity with Neural–PI controllers parameterized through strictly convex neural networks. This structure provides universal approximation capability, facilitates the inclusion of communication constraints, and allows neural storage functions to serve as Lyapunov functions, thereby ensuring stability and zero steady-state tracking error. While the reported results demonstrate improved transient and steady-state performance in traffic and power network applications, the reliance on passivity-based methods can be conservative, potentially leading to slower dynamic responses in highly stochastic microgrids, and unstructured neural networks may still introduce sensitivity to modelling accuracy.

Similarly, the framework proposed in [76] exploits input–output dissipativity by learning neural storage and supply rate functions to achieve both stability and optimality guarantees for nonlinear systems. Although effective in safety-critical control applications, this approach often requires extensive data and computational resources, and maintaining dissipativity properties in practice can be challenging. Furthermore, while stability is assured, optimisation of long-term performance metrics such as cumulative energy usage may remain limited, since training is typically sample-based rather than trajectory-based.

To address these limitations, this paper proposes a Neural–PI-based adaptive bound adjustment strategy that not only guarantees closed-loop stability but also targets optimal performance over an infinite horizon. By integrating learning-based adaptation with event-triggered control, the proposed framework enables real-time bound refinement that more accurately reflects current system conditions, improves responsiveness to disturbances, and achieves a better balance between performance and efficiency.

### Proposed online switching load frequency control method

Significant research gaps remain in effectively integrating advanced control strategies with the unique characteristics of islanded microgrids, particularly those with high penetration of variable renewable energy sources such as solar and wind, where variability, limited spinning reserves, and low inertia pose serious stability challenges [77]. Additional unresolved issues include the real-time deployment of computationally intensive algorithms under low-latency communication constraints [78], insufficient optimisation of spinning reserves using technologies such as energy storage systems and virtual synchronous generators [79], and the difficulty of decentralized coordination among multiple distributed energy resources in complex microgrid architectures [80]. Addressing these gaps is essential to improve reliability, stability, and efficiency as microgrids evolve toward renewable-dominated energy systems, with event-triggered control approaches increasingly recognized as promising solutions for balancing performance and resource efficiency [81, 82].

In response, this paper proposes an online event-triggered load frequency control switching framework tailored for islanded microgrids with limited spinning reserves and high renewable penetration. The proposed approach integrates a dynamic power system model, real-time inertia estimation, predictive control, and a supervisory online switching algorithm that selects among pre-trained Neural-PI controllers corresponding to different inertia modes. By enabling adaptive bound adjustment through machine learning, the framework overcomes the limitations of bounded adaptive event-triggered mechanisms, reducing unnecessary control actions, communication overhead, and energy consumption while maintaining frequency stability under uncertainty.

In the Online Event-Triggered Switching LFC, an event-triggered scheme is employed to reduce unnecessary communication and computation compared to fixed-time sampling. Under event-triggered control, frequency measurements or controller updates occur only when a threshold is exceeded, rather than at every clock tick. This is attractive in islanded microgrids because small deviations (e.g. during normal operation) require no action, greatly lowering communication traffic. In contrast, periodic sampling wastes bandwidth on minor fluctuations, and self-triggered approaches add predictive overhead. Event-triggered control reduces average communication by eliminating needless updates, and maintains stability via threshold design. Periodic control has the simplest implementation but highest overhead. Self-triggered control can save communication but requires extra computations and relies on accurate modeling. For islanded LFC with variable inertia, event triggering is often chosen because it reacts directly to actual frequency deviations and can adapt its pacing, while providing formal stability guarantees (via dwell-time or ISS arguments) similar to periodic schemes. Event triggers still guarantee stability (with dwell-time conditions) while improving responsiveness to large disturbances. The tradeoff is slightly more complexity (monitoring conditions, trial‐phase computations). Overall, event triggering yields dramatically lower average message rates and compute loads, with conservative worst‐case delays only when needed.

The proposed OETC strategy also exploits the data-driven adaptability of machine learning to capture complex microgrid dynamics and respond effectively to rapid load and generation fluctuations without continuous control signaling. This results in fewer corrective events, improved utilization of scarce spinning reserves, and enhanced robustness against renewable intermittency. Although the proposed event-triggered Neural-PI controller enhances communication efficiency and dynamic performance, it is inherently vulnerable to cyber threats such as false data injection (FDI) attacks targeting measurement channels. Such attacks can corrupt both the triggering mechanism and control input signals, potentially leading to degraded or unstable system behavior. To address this limitation, recent studies propose observer-based and detection-triggered mitigation frameworks, where compromised measurements are reconstructed and filtered before control action. Incorporating such mechanisms into the proposed framework would enhance its cyber-resilience by ensuring that the event-trigger condition and Neural-PI controller operate on trusted state estimates rather than raw measurements. The key contributions of this work include the development of a novel Neural-PI controller that combines learning capability with classical control structure, demonstration of its effectiveness for load frequency control in islanded microgrids, and validation of its adaptability and robustness under changing operating conditions and system dynamics.

Consequently, a comparative evaluation of the proposed approach against traditional and advanced LFC methods is presented in Table 5.

**Table 5:** Table of comparison between the proposed method, traditional and advanced methods

LFC methods	Response time	Adaptability	Handling non-linear sys parameter control	Parameter control	Scalability	Handling complex networks	Comms dependence & cited literature
Traditional	Slow	Low	Poor	Fixed	Limited	Struggles	High; [28, 30, 31, 46]
Advanced	Fast	Medium	Good	Adaptive	Moderate	Handles some	Medium; [45, 53–55, 59],
Hybrid	Fast	Moderate	Good	Hybrid	Moderate	Handles some	Medium; [1, 12, 21, 56, 60, 63, 64]
Proposed	Fast	High	Excellent	Self/Robust	High	Handles well	Low-Medium

### Paper organization

This subsection presents the organization of the paper, which is structured into five sections. Section I introduces the study by outlining the motivation, reviewing related literature, identifying research gaps, and highlighting the main contributions. Section II describes the methodology, including the proposed LFC approach and the underlying theoretical framework. Section III presents the numerical simulations and results, covering the case studies, base controllers, and the benchmark PID controller. Section IV discusses and analyses the results, while Section V concludes the paper by summarizing the key findings and contributions.

## Imethodology

### Explanatory framework for the proposed load frequency control

**Fig. 2:**
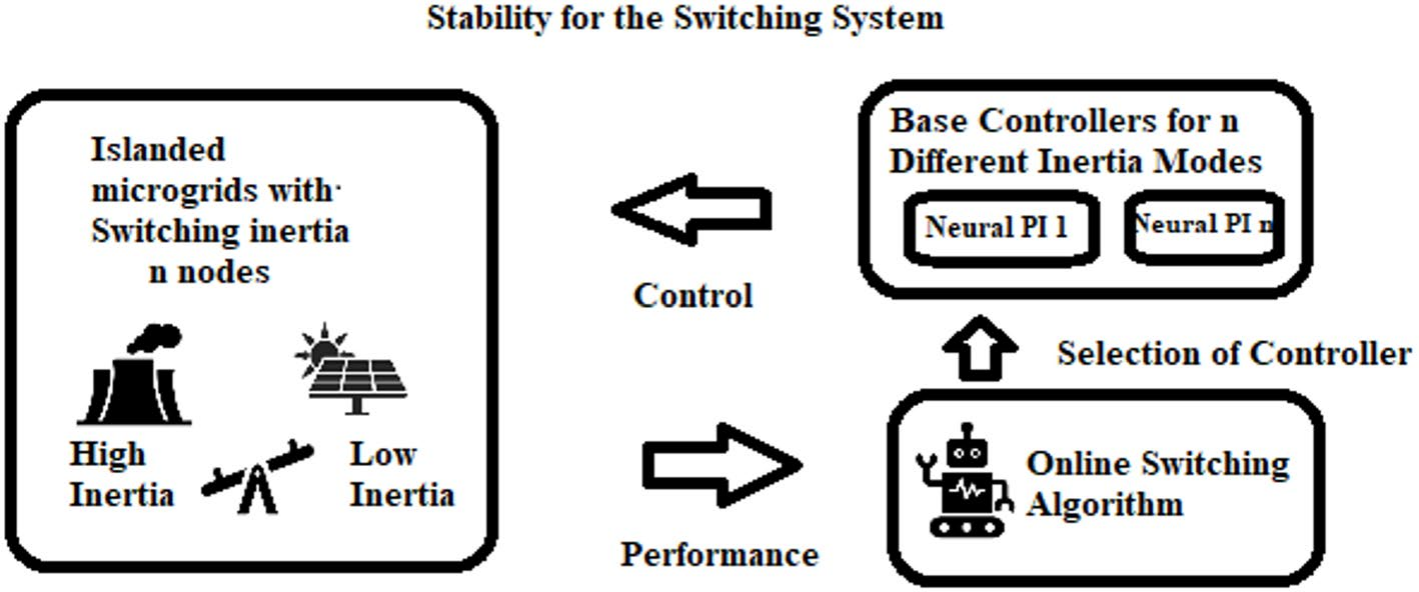
Conceptual framework of the online switching LFC method

The proposed online event-triggered LFC switching framework is illustrated in Fig. 2 through a stability-oriented block diagram. The diagram is organized into three main sections. The left-hand block represents the islanded microgrid (MG) with *n* nodes exhibiting switching inertia, a condition that arises from the high penetration of renewable energy resources (RERs). Each node possesses distinct inertia characteristics, which collectively influence the system’s dynamic response to disturbances and load variations. Thus, Fig. 2 presents a schematic illustration of the proposed online event-triggered load frequency control (OETC) switching mechanism developed in this study.

The lower right-hand block corresponds to the online switching algorithm, which incorporates a supervisory layer and operates with an unbounded adaptive switching threshold. This supervisory layer continuously monitors the inertia-dependent performance of the islanded MG, evaluates the system’s operating state in real time, and determines whether control updates are required. The inertia of individual nodes may vary between high and low states relative to the prevailing load demand, and these variations are tracked by the algorithm using both current and historical system data, without reliance on future state information.

The upper right-hand block represents the base controller, which comprises a bank of pre-trained Neural-PI controllers designed for different inertia modes. Based on the inertia state identified by the online switching algorithm, the appropriate Neural-PI controller is automatically selected to apply corrective control actions and restore frequency stability. The Neural-PI controllers combine the learning capability of neural networks with the robustness and simplicity of conventional PI control. Specifically, the neural component learns and adapts to the nonlinear and time-varying dynamics of the MG, while the PI component regulates frequency by adjusting generator outputs to ensure accurate and stable operation.

Overall, the framework formalizes the dynamic interactions of distributed energy resources, energy storage systems, and variable loads within an islanded MG. By employing an unbounded adaptive event-triggered control strategy, the proposed approach reduces unnecessary control actions, lowers communication and actuation burden, and minimizes wear on system components. Consequently, the framework achieves a balanced trade-off between system stability and efficient resource utilization by adaptively tuning thresholds and control parameters in accordance with real-time operating conditions.

### Theoretical framework of the proposed method

The frequency instability arising from the bounded nature of existing ETC schemes [62] highlights the challenge of tuning adaptive thresholds, as excessively narrow bounds limit adaptability while overly wide bounds can induce oscillations and disturbances. To address this, the proposed OETC switching framework enhances stability and robustness by dynamically switching controllers based on real-time system states, disturbance levels, spinning-reserve availability, and communication conditions. The approach adopts a hybrid control architecture that integrates machine-learning-based prediction, real-time inertia estimation, and Neural-PI controllers to ensure effective frequency regulation under stochastic operating conditions. The theoretical framework therefore comprises dynamic power system modelling, online event-triggered switching, Neural-PI control design, real-time inertia estimation with predictive control, stability guarantees, and an event-triggered switching algorithm. Thus,

- *Dynamic power system modelling*

The islanded microgrid is framed as a network of interconnected nodes, as represented in Eq. (1).

1 $$\:G=\left(V,E\right)$$

where: V - Set of buses, E - Transmission lines connecting the buses, i - Bus, $\:{\delta\:}_{i}\left(t\right)$ - Phase angle deviation of each bus, $\:{\omega\:}_{i}\left(t\right)$ - Frequency deviation. Hence, Eqs. (2) and (3) are the equations for the dynamics of the system.

2 $$\:{\delta\:}_{i}={\omega\:}_{i}-\frac{1}{n}\sum_{j=1}^{n}{\omega\:}_{j}$$

3 $$\:{M}_{i}\left(t\right){\dot{\omega\:}}_{i}={P}_{i}-{D}_{i}{\omega\:}_{i}+{u}_{i}-\sum_{j\in\:{N}_{i}}{B}_{ij}\mathrm{sin}\left({\delta\:}_{i}-{\delta\:}_{j}\right)+\varDelta\:{d}_{i}$$

where: $\:{M}_{i}\left(t\right)$ – Time-Varying inertia of bus i, which changes dynamically with the status of the synchronous generators, $\:{D}_{i}$ – Damping coefficient from loads and generators, $\:{u}_{i}$ – Control action at bus I, $\:{\varDelta\:d}_{i}$ – External disturbances such as load changes or renewable power fluctuations.

For the sake of reflecting real-world variability, the time-varying inertia $\:{M}_{i}\left(t\right)$ is modeled as switching based on the different inertia modes in which the system operates depending on the availability of generators and renewable resources. This variability requires control design to take different approaches for real-time adaptation.

- *Online event-triggered switching framework*

The proposed control design uses a dynamic event-triggered switching mechanism, allowing for real-time selection of one of many possible pre-trained controllers depending on the present state of the system. Each controller within the pool is optimised for specific operating conditions-dominantly high-inertia or low-inertia scenarios. The control action $\:{u}_{i}$ is defined as:

4 $$\:{u}_{i}={\pi\:}_{i}({\omega\:}_{i},{s}_{i},{\left\{{s}_{j}\right\}}_{j\in\:{N}_{i}})$$

where: $\:{\pi\:}_{i}$ – Control policy for bus I, comprising proportional and integral terms, $\:{s}_{i}$ – Integral state capturing cumulative frequency deviations, $\:\left\{{s}_{i}\right\}$ – States of neighboring buses j.

A supervisory layer continuously monitors system performance and determines the control mode by triggering a switching event whenever frequency deviation exceeds a predefined threshold.

5 $$\:{\parallel\omega\:\left(t\right)\parallel}_{\infty\:}>\epsilon\:$$

where: $\:\epsilon\:$ – An adaptive threshold which adjusts dynamically based on system stability and operating conditions.

- *Neural-Pi control design*

Each controller employs a Neural-PI structure, combining: A proportional term parameterized as a monotonic neural network which ensures non-linear adaptability to system disturbances.

6 $$\:{\pi\:}_{i}^{\left(p\right)}\left({\omega\:}_{i}\right)={\omega\:}^{T}ReLU(W{\omega\:}_{i}+b)$$

A linear integral term to ensure steady-state frequency restoration which ensures exponential input-to-state stability for the closed-loop system.

7 $$\:{\pi\:}_{i}^{\left(i\right)}\left({s}_{i}\right)=k{s}_{i}$$

- *Real-time inertia estimation and predictive control*

The framework embeds real-time inertia estimation to allow accurate assessment of the system’s operating state. Predictive algorithms may analyse the sensor data to estimate the inertia fluctuations and forecast disturbances. The supervisory layer uses this information to select pre-trained controllers suited for the most likely operating conditions such that rapid and robust performance would be guaranteed. In addition, reinforcement learning (RL) based optimisation is used to increase the performance of controller selection. The RL agent evaluates the historical data of the system and learns an optimal switching policy in order to minimize frequency deviations and control costs.

- *Stability guarantees*

The switching framework enables stability through exponential input-to-state stability. For the system state vector $\:x\left(t\right)$, stability is guaranteed by:

8 $$\:{\parallel x\left(t\right)\parallel}_{2}\le\:{k\rho\:}^{t}{\parallel{x}_{0}\parallel}_{2}+\beta\:{\parallel\varDelta\:d\left(t\right)\parallel}_{\infty\:}$$

where $\:k,\rho\:,\beta\:$ are constants, thus, the system is guaranteed to be bounded in the presence of disturbances and recovers to its equilibrium at an exponential rate after the disturbances are removed.

- *Event-triggered switching algorithm*

Three phases are recognized in the switching algorithm:

#### Selection Phase

All candidate controllers are evaluated for a period, however brief, yielding a cost for each.

9 $$\:\:\:\:\:\:\:\:\:\:\:\:\:\:\:\:\:\:{J}_{i}=\frac{1}{T}{\int\:}_{0}^{T}(\frac{1}{2}{c}_{i}{u}_{i}^{2}+\lambda\:({\parallel\omega\:\parallel}_{2}^{2}+{\parallel\omega\:\parallel}_{\infty\:})dt)$$

#### Trial Phase

Deploy the most promising controller for a fixed time period, monitoring its performance.

#### Deployment Phase

Implement the selected controller until the next trigger from the event.

The chosen controller selection probabilities, as engineered in this algorithm, evolve dynamically via a multi-arm bandwidth optimisation framework, assuring highly efficient and adaptive designs through the control decisions. The event-trigger threshold ε critically trades off performance against communication load. A smaller ε (e.g. 0.01 Hz) yields fast frequency correction (low overshoot/settling) but triggers many events (high switching frequency and comms), while a larger ε (e.g. 0.1 Hz) conserves bandwidth at the cost of slower response and larger deviation. This paper implicitly uses a fixed ε (unspecified) to enter the controller selection phase. However, to ensure stability, ε must respect the dwell-time assumptions of the ISS proof: too frequent triggers could violate slow-switching conditions. Adaptive threshold schemes (e.g. hysteresis, event-rate controllers, and variance-based updates) can dynamically balance this trade-off. For example, one may increase ε when events become too frequent, or use a continuous adaptation ODE. Implementation-wise, thresholds affect average/peak message rates (smaller ε → near-periodic updates), computation (more trials), and robustness. Mitigations include minimum inter-event times, hysteresis bands, and filtering.

**Fig. 3:**
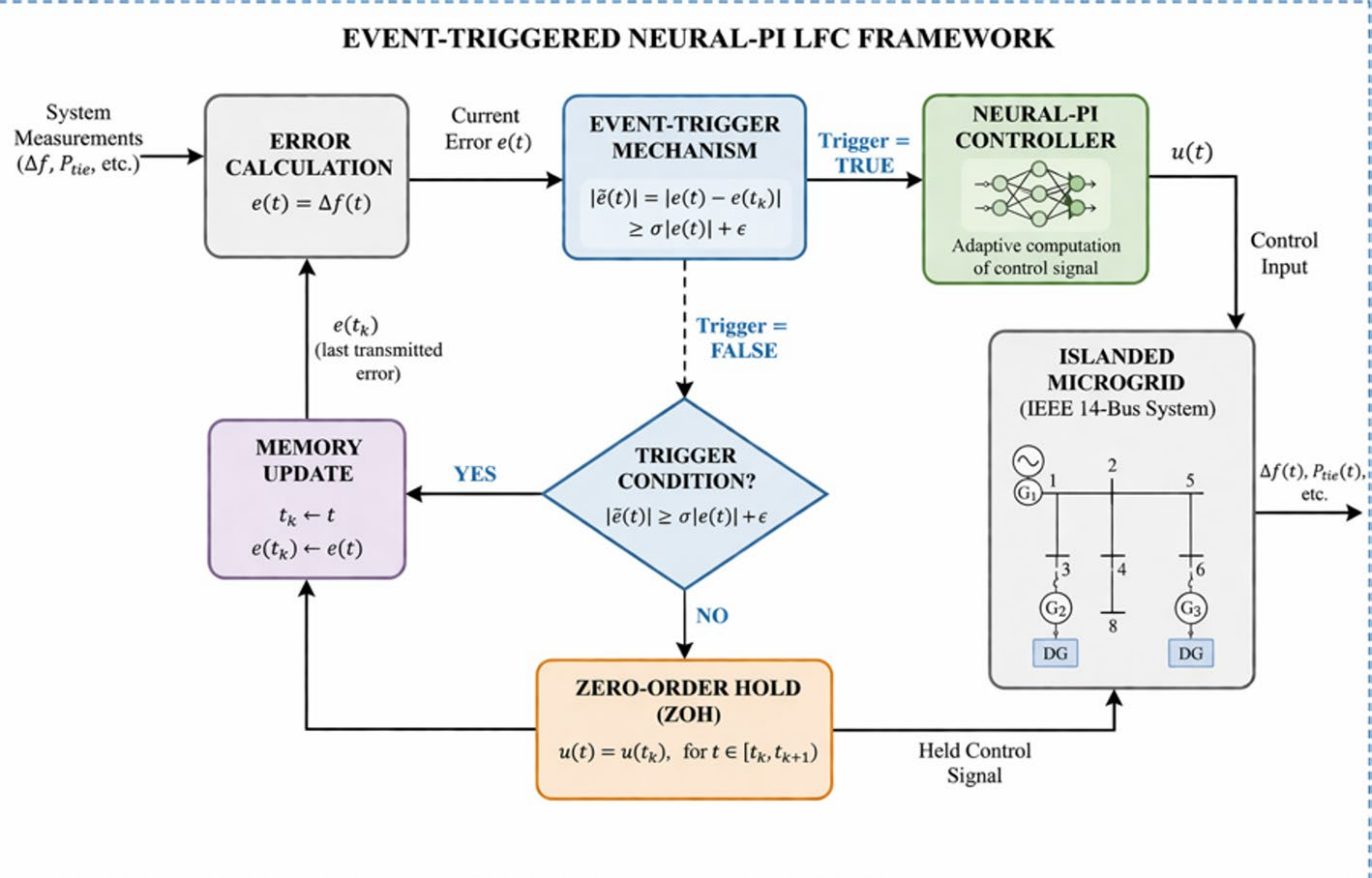
Conceptual Event-Triggered Neural-PI LFC Framework of the online switching LFC method

The event-trigger mechanism is implemented by monitoring system error continuously, comparing it with the last transmitted value, updating control only when deviation exceeds a threshold and holding control otherwise, as depicted in Fig. 3. This is to ensure efficient communication, reduced computation and maintained stability.

## Numerical results and simulations

### Case study

The proposed framework is validated through simulations on an islanded microgrid with high renewable RER penetration using Python within a Jupyter Notebook environment. Jupyter Notebook is adopted for its strong support for modern machine-learning workflows, interactive code execution, real-time visualization, and access to Python’s open-source scientific libraries, making it well suited for data-driven control studies and rapid prototyping. The Neural-PI controller is developed and tested through iterative training, adaptive threshold adjustment, and inline visualization, enabling efficient implementation and debugging of the event-triggered switching logic. The microgrid is dynamically modelled as a graph-based network using *NetworkX* and *SciPy*, with system parameters derived from the IEEE 14-bus test system, allowing the evaluation of frequency stability under varying inertia, renewable intermittency, and load disturbances. The simulation results demonstrate the framework’s effectiveness in maintaining frequency stability while reducing communication overhead and control cost compared with conventional fixed-parameter control approaches. The proposed Neural-PI event-triggered LFC framework is inherently scalable due to its event-driven communication mechanism, which significantly reduces unnecessary data transmission, and its adaptive neural control structure, which can handle nonlinearities in large-scale systems. While the current study validates the approach using the IEEE 14-bus system, similar performance improvements—such as reduced communication burden and enhanced frequency regulation—are expected in larger systems, particularly when implemented in a distributed control architecture. However, scalability may require additional considerations such as decentralized coordination, retraining of neural networks, and communication delay compensation. Future work will extend the proposed framework to larger benchmark systems and interconnected microgrid environments.

**Fig. 4:**
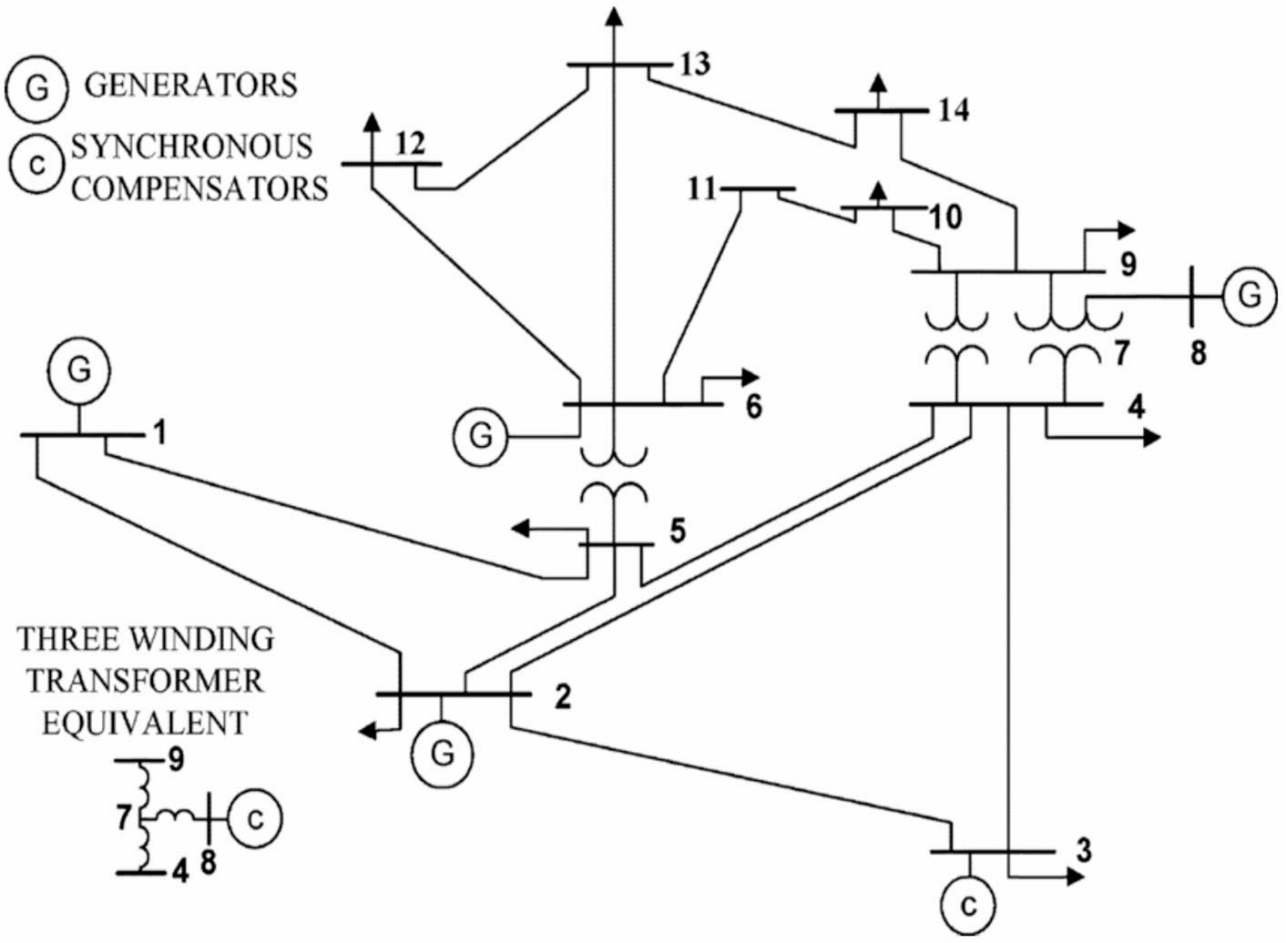
Single line diagram of the IEEE 14-Bus system [77]

The IEEE 14-bus system is implemented by first solving the steady-state power flow to obtain the operating point, followed by dynamic modeling of generator, turbine, and governor dynamics. The proposed event-triggered Neural-PI controller is then integrated into the system to regulate frequency deviations under load disturbances. The data used to validate this study were generated synthetically through simulations due to the impracticality of conducting experiments on a real microgrid system. The data acquisition process followed three main stages. First, a dynamic islanded IEEE 14-bus (Fig. 4) microgrid was modelled in Python using admittance matrices to represent network topology, while generator inertia, damping coefficients, and disturbances were probabilistically defined to emulate renewable intermittency and load variability. Multiple inertia modes (low, nominal, and high) were incorporated and switched in response to event-triggering conditions. Second, transient disturbances were algorithmically injected to replicate real-world operating volatility arising from sudden load and renewable power fluctuations. Third, synthetic training datasets were generated for the Neural-PI controllers, where frequency deviation served as the input and control actions were derived as inertia-scaled responses to enforce proportional control behaviour. System responses, including frequency deviation and phase-angle trajectories, were computed via numerical integration of the governing differential equations and evaluated using predefined event-triggering thresholds to validate controller switching decisions.

The simulations leveraged standard MATLAB-based power system datasets, including *IEEE_14bus_Kron.mat*,* Kron_14_cut_2line.mat*, and *Sol_linear.mat*, which were adapted for use within the Python environment. The IEEE_14bus_Kron.mat dataset provided the benchmark system parameters and network characteristics for baseline analysis. The *Kron_14_cut_2line.mat* dataset, obtained through Kron reduction and intentional transmission line removal, enabled robustness testing under faulted and reduced-network conditions. The *Sol_linear.mat* dataset supplied a linearized state-space model used as a performance reference for comparing conventional linear controllers with the proposed Neural-PI approach in terms of key metrics such as settling time and overshoot. Collectively, these datasets facilitated reproducible and scalable evaluation of the proposed framework, demonstrating its robustness, adaptability, and performance advantages under diverse operating conditions.

The adaptive Neural-PI controller’s stability is guaranteed by Lyapunov/ISS analysis and careful controller design. This paper proves that each fixed‐inertia mode with a Neural‐PI has exponential input-to-state stability (Exp-ISS). Accordingly, a custom Lyapunov function shows that frequency deviations decay exponentially (with a bounded disturbance gain) under the monotonic Neural‐PI architecture. Importantly, the proportional part is a stacked ReLU NN with strictly positive slopes (“monotonic”), and an integral term ensures zero steady‐state error. These properties make the system dissipative: negative Lyapunov derivative is achieved because the monotonic nonlinearity always dissipates energy. Under switching, a common Lyapunov function and an average dwell‐time condition (inertia switches much slower than control) extend Exp‐ISS to the switched. Assumptions include bounds on disturbances, sufficient damping, and exact measurements. The result is that as long as frequency excursions remain below threshold, the Lyapunov function ensures stability. In practice, this is enforced by choosing conservative thresholds, adding hysteresis (to avoid chattering), and ensuring controllers operate within actuator limits. Robustness to noise and delays is not explicitly proved but can be handled with fallback strategies (hold last controller if communications fail). Overall, the architecture and analysis guarantee global exponential stability for each mode and under slow mode switching.

### Base controllers (Neural-PI controllers)

The proposed framework is implemented using a set of pre-trained base controllers composed of multiple Neural–PI controllers designed to address frequency regulation under dynamically varying system inertia. The simulation integrates reinforcement learning and deep learning techniques, implemented using TensorFlow and Keras within a custom-built power system simulation environment. The implementation begins with modelling grid dynamics, followed by the formulation of control actions and the development of neural controllers capable of learning optimal strategies for maintaining frequency stability. The simulation environment accurately represents power system behaviour by incorporating generator inertia, damping coefficients, communication constraints, and power mismatches, enabling realistic state transitions and control interactions under disturbance conditions.

At the core of the control strategy is a Neural–PI controller implemented as a minimal recurrent neural network cell. The proportional component is realised through monotonic neural networks with *ReLU* activations to handle nonlinear system dynamics, while the integral component employs linear structures to ensure steady-state frequency restoration. This hybrid architecture balances adaptability and stability, allowing effective response to transient disturbances while maintaining long-term regulation accuracy. Reinforcement learning is employed to train the controllers through continuous interaction with the simulated environment, where control policies are optimised to minimise frequency deviations and control effort.

Stability is reinforced through embedded mathematical constraints, including exponential input-to-state stability conditions, ensuring robustness against disturbances. Data preprocessing routines initialise system parameters such as inertia, damping, and network connectivity to support accurate simulation and effective training. Beyond load frequency control, the developed framework is applicable to broader power system optimisation and dynamic system modelling tasks, providing a flexible platform for testing and refining adaptive control strategies in realistic grid scenarios. A description of how the Neural-PI controller are trained as well as the dataset and operating scenarios used to ensure generalization across different microgrid conditions are presented subsequently.

The Neural-PI controllers are trained using reinforcement learning. A recurrent neural network (RNN) framework incorporates the power system dynamics into the training loop. Notably, at each time step the RNN cell takes the current grid state (all bus frequency deviations) and outputs a control action. The power system model then propagates to the next state, which feeds into the next RNN step. The loss function is the cumulative cost over a short horizon (like 3 s) combining error and control effort. Training uses gradient descent to minimize this loss over many randomized disturbance trajectories. A learning rate scheduler (decay every episodes) is applied. Training runs with each base controller converging in on the other of minutes. Each base controller is trained under a fixed inertia scenario. For each inertia modes (low, medium, high inertia), training episodes of 3 s duration are generated. At a random time within each episode, a random step change in net load is applied. The RNN is unrolled for the 3 s trajectory (with timestep Δt ~ 0.01 s), and the policy parameters are updated to minimize the total frequency cost. This approach ensures closed-loop stability by design.

### Simulation models anddataset generation

Training and testing use a high-fidelity dynamic model of an islanded microgrid (the IEEE 14-bus). Generators are modeled with swing equations and standard governor dynamics; inverter-interfaced resources (renewables, batteries) provide control via set-point adjustments. Typical parameters (damping coefficients, time constants, power limits) are drawn from standard data for the 14-bus system. Inertia is varied to simulate different microgrid mixes. Three representative inertia constants were used: H = 0.3 (very low inertia, mostly inverter-based), H = 1.0 (nominal), and H = 5.0 (high inertia). Damping factors and governor time constants are fixed at nominal values (from the IEEE-14 data) but could also be randomized. The Neural-PI controllers are trained under each H-value separately. The primary disturbances are net load steps. Each training episode includes one random step change in total load (e.g. ±X MW), applied at a random time in the 3 s window. The magnitude of load steps is drawn from a range (e.g. ±10–30% of base load) to cover mild and large events. In some tests, two disturbances occur (e.g. at t = 5 s and t=15s in a 20 s scenario). Renewable variability can be mimicked by superposing random fluctuations or by interpreting net-load steps as equivalent renewable output changes. The state is sampled at high rate (e.g. 100 Hz, Δt ≈ 0.01 s). Training uses thousands of episodes across modes. (Exact counts aren’t published, but training times suggest on the order of 10^4–10^5 samples.) There is no separate “dataset” split in the usual sense; instead, controllers are trained until convergence and then validated on separate simulation runs. In one evaluation 100 random test trajectories (3s each) were run in each inertia mode to compute average costs. To ensure generalization, many variables are randomized: disturbance timing, disturbance size, initial conditions, and inertia-transition sequences. Crucially, inertia is switched unpredictably in the test scenarios: at 5 s intervals the system may jump between H = 0.3, 1.0, or 5.0 according to preset probabilities. This mimics microgrids with varying online resources. Randomized training (e.g. random step times) ensures the Neural-PIs see a wide range of conditions. Operating scenarios is as shown at Table 6 below.

**Table 6:** Operating Scenarios

Scenario	Setup/Purpose	Parameters	Expected controller behaviour
Low Inertia	Heavy inverter-penetration case. Test fast dynamics under large disturbance	Inertia H = 0.3; one step load change (+ΔP at t ≈ 1 s)	Frequency deviates quickly; controller must act aggressively. Neural-PI-0.3 should be used (it’s tuned for fast response)
Medium Inertia	Typical grid. Test moderate dynamics	H = 1.0; one load step at t≈1s	Moderate frequency drop; a balanced response. Neural-PI-1.0 expected to perform best
High Inertia	Synchronous-heavy grid. Test slow dynamics and larger energy buffer	H = 5.0; one load step at t≈1s	Frequency drift is slower; larger control efforts tolerated. Neural-PI-5.0 should excel (others may overshoot)
Frequent Switching	Test switching logic. Inertia randomly jumps mid-way through simulation	H transitions every 5 s (e.g. 0.3→1.0→5.0), random steps at 5 s,15s	Initial controller matched to H. On each change, switching algorithm should detect suboptimal performance and switch to the pre-trained controller for the new H (via trial phase)
Renewable surge/fall	Simulate variable generation. Load changes mimic cloud/ramp events or wind gusts	Repeated ± step changes or ramps over 20s	All controllers must adapt; switching may help if generation change also implies inertia shift. The Neural-PI’s feedback (with appropriate P gain) will stabilize frequency
Parameter uncertainty	Ensure robustness. Randomize secondary parameters (damping, time-const) in test	Damping ζ varied ± 20%; governor time-const varied	Controllers should still stabilize despite parameter mismatch. Using monotone P ensures some robustness. May reveal if extra domain randomization is needed
Noisy Measurement	Add random noise to frequency feedback	White noise on Δf (± 0.01 Hz)	Expect slight performance degradation. Filter/hysteresis may be needed

### PID controller

Neural–PI controllers extend conventional PID control by integrating neural networks that adaptively tune controller parameters in response to changing system dynamics. To validate the effectiveness of the proposed hybrid Neural–PI controller relative to a traditional PID controller, comparative simulations are conducted using a standard PID design [57]. The evaluation focuses on key performance indices, including response time, stability, and robustness under varying operating conditions.

A dedicated Python-based simulation framework, employing the Control, NumPy, and Matplotlib libraries, is used to assess both controllers. The PID controller is first designed with fixed proportional, integral, and derivative gains, and its transient and steady-state responses to step inputs are analysed. Subsequently, the Neural–PI controller is implemented within a learning framework using TensorFlow, where the PI control law is represented by a neural network whose parameters are adaptively updated based on observed system behaviour. The network is trained using supervised or reinforcement learning informed by prior PID performance.

Comparative simulations are then carried out, and the system responses under PID and Neural–PI control are analysed. Quantitative performance metrics, including the root mean square error (RMSE) and the integral of time-weighted absolute error, are used to demonstrate the improved dynamic performance and robustness achieved by the Neural–PI controller compared with the conventional PID approach.

## Result analysis and discussion

### Analysis of result

**Fig. 5:**
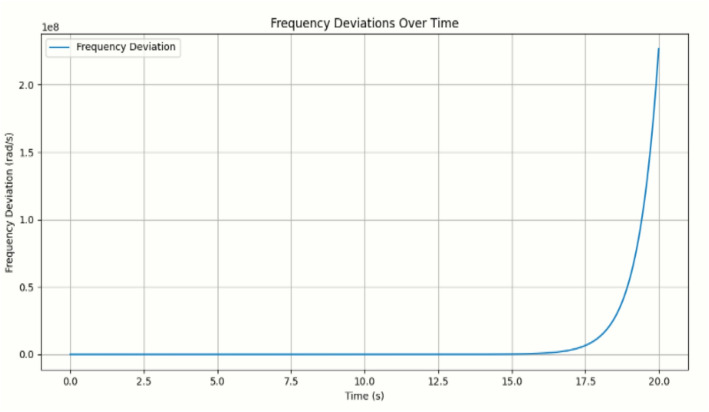
Plot of frequency deviation over time

**Fig. 6:**
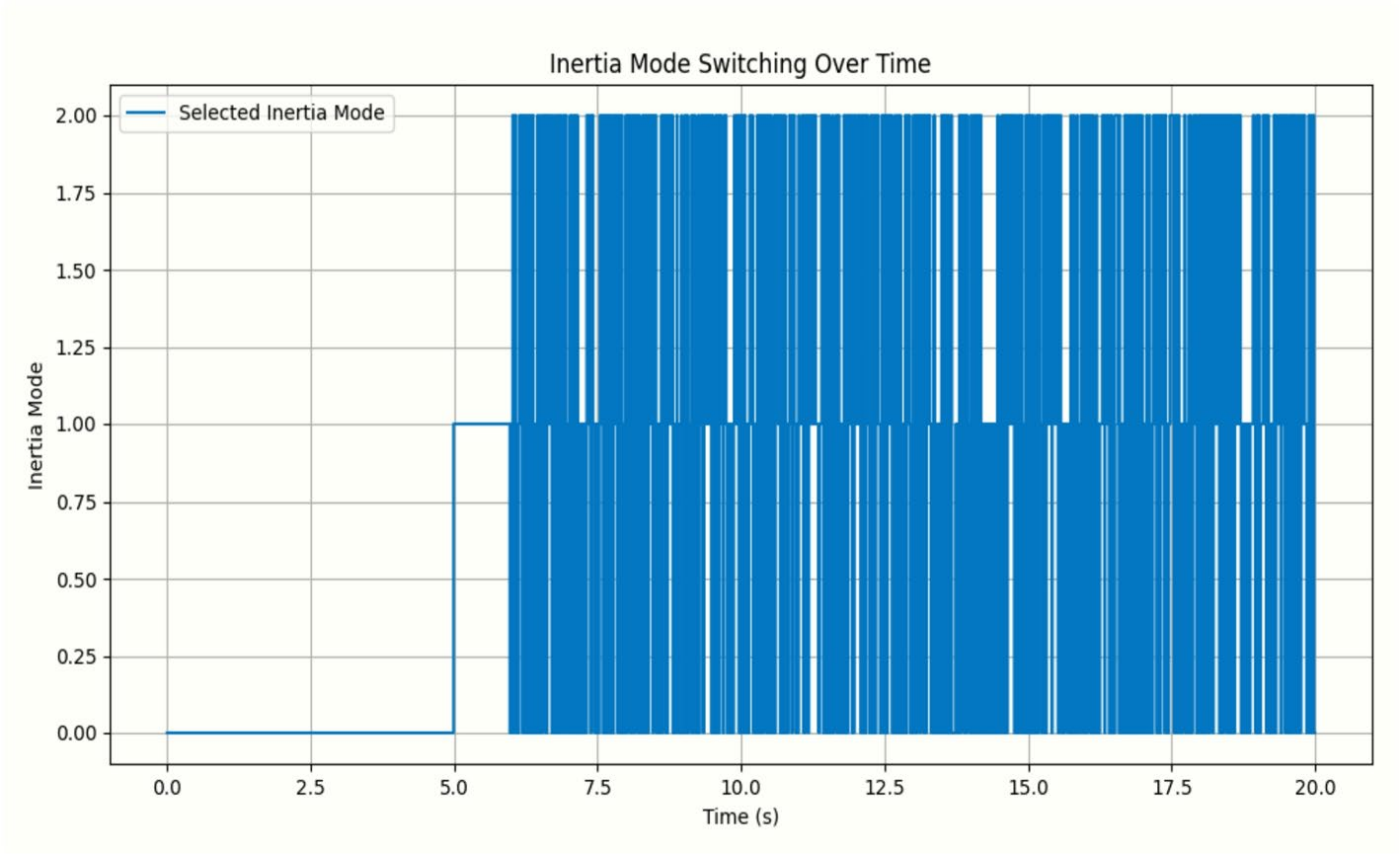
Plot of inertia mode switching over time

**Fig. 7:**
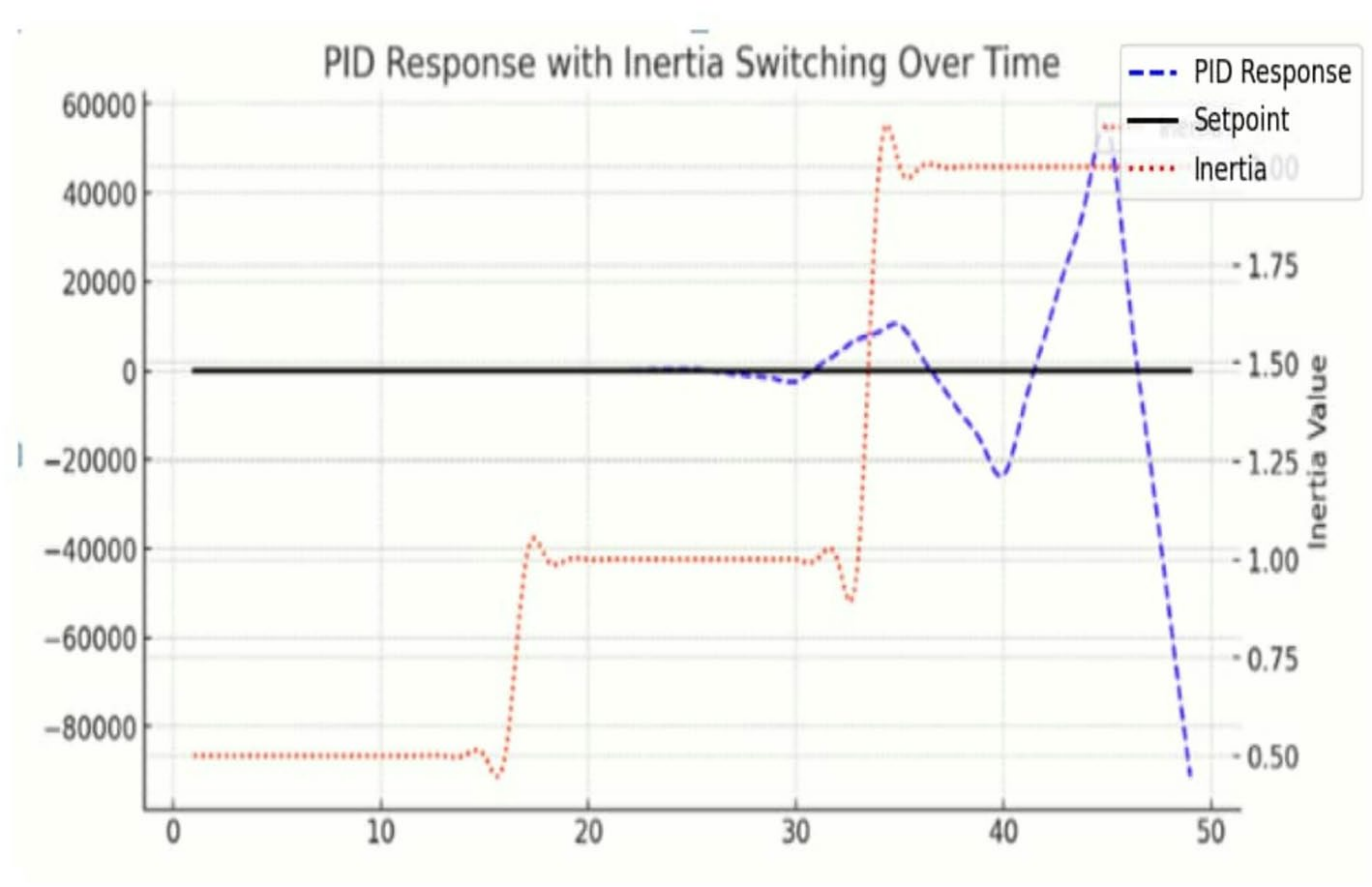
Plot of proposed method and PID response with inertia switching over time

The simulation results obtained using Python in a Jupyter Notebook environment are presented in Figs. 5, 6 and 7. The proposed online event-triggered switching load frequency control framework employs a Neural–PI controller to achieve an optimised dynamic response. The simulation begins with the mathematical modelling of power system dynamics, where differential equations describe frequency deviations and time-varying inertia. Event-triggering conditions are then incorporated to determine when control actions are executed, striking a balance between control efficiency and system performance by activating control only when necessary to preserve stability.

The developed Python script, based on the IEEE 14-bus system with dynamic inertia modelling, generates the frequency deviation trajectories shown in Fig. 5 and the corresponding inertia-mode switching profiles illustrated in Fig. 6. Time-varying disturbances and inertia transitions are introduced, and the event-triggered switching mechanism adaptively selects appropriate control actions to maintain frequency stability. The study evaluates system behaviour under different inertia modes and demonstrates the ability of the adaptive strategy to regulate frequency despite changing operating conditions.

The simulation is initialised using key parameters summarised in Table 7, including a 14-bus network, three inertia modes, and a total of 2000 simulation time steps. The inertia modes represent distinct stability levels, defined as low (0.3), nominal (1.0), and high (5.0), enabling a systematic assessment of controller performance across varying system inertia conditions.

A discrete time step of 0.01 s was adopted to enable high-resolution monitoring of system dynamics. The *create_system()* function was used to generate the network admittance matrix, which captures electrical interactions among buses. This matrix was randomly initialized to emulate realistic grid conditions, with appropriate adjustment of diagonal elements to ensure a physically valid power-flow representation. Arrays for frequency deviation (ω) and phase angle (δ) were also defined and initialized to zero, providing a stable initial operating point.

The Neural–PI controller was implemented via the *create_neural_pi_controller()* function using the TensorFlow–Keras framework. The controller architecture consists of a three-layer neural network, with a single input representing frequency deviation, followed by two hidden layers of 20 neurons each employing ReLU activation functions, enabling effective nonlinear approximation and adaptive control behaviour.

**Table 7:** Simulation parameters

Parameters	Numbers	Remarks
No of Bus	14	14-Bus power system
No of Inertia Modes	3	Low (0.3). Standard (1.0) and High (5.0)
Total Time Steps (s)	2000	Discrete time step of 0.01 s.

The output of the Neural-PI controller produced a single linear value representing the control action and was compiled using an Adam optimiser with an MSE loss function to ensure effective learning. Multiple Neural-PI controllers were trained for different inertia levels using synthetic input-output data, where inputs *(x_train)* ranged from − 1 to 1 and outputs *(y_train)* were calculated as the negative product of inertia and input, representing a proportional control response. Each controller was trained for 10 epochs to capture the system dynamics.

An event-triggered switching mechanism was implemented through the *event_triggered_switching()* function, which monitored the maximum absolute frequency deviation (ω). If ω exceeded 0.01 rad/s, the system switched to a different inertia mode probabilistically, providing flexibility in response to disturbances. The main simulation loop, contained in *simulate()*, tracked the temporal response of the system while updating inertia modes according to predefined schedules (low: 0–5 s, normal: 5–10 s, high: >10 s) and random disturbances (e.g., 5–5.5 s and 10–10.5 s). Frequency deviations and their rates of change were computed at each step using the swing equation, considering the effects of the admittance matrix, phase angles, inertia, and injected disturbances.

The event-triggered switching mechanism allowed the control system to adapt dynamically, maintaining stability by switching between inertia modes in response to real-time deviations. Simulation data including time, frequency deviations, phase angles, and inertia modes were collected for analysis, with results presented in Figs. 5 and 6 and frequency deviations summarized in Table 8. Comparisons were made between traditional PI control, continuous control, and the proposed event-triggered Neural-PI approach. The simulations demonstrated that the Neural-PI controller, combined with event-triggered switching, effectively mitigates disturbances, stabilizes frequency, and provides an adaptive, intelligent control strategy under varying operating conditions.

**Table 8:** Microgrid frequency deviation with time

Frequency deviation (rad/s)	Time (s)
0.0	0.00
0.0	2.50
0.0	5.00
0.0	7.50
0.0	10.0
0.0	12.5
0.0	15.0
0.1	17.5
2.0	20.0

The frequency deviation graph in Fig. 5 illustrates the dynamic behaviour of the system under different operating conditions. From 0 to approximately 15 s, frequency deviations remain close to zero, indicating stable operation, where the selected pre-trained Neural-PI controllers successfully maintain the microgrid at nominal frequency. At this stage, the system does not require additional inertia compensation, and the controllers operate in a stable monitoring mode.

A significant transition occurs around 17.5 s when the system switches to a high-inertia mode (inertia factor 1.0) to dampen frequency deviations and recover from instability. During a 2.5 s interval, frequency deviations reach 0.1 rad/s, prompting the Neural-PI controllers to adjust control signals adaptively. In a more extreme case, deviations increase sharply from 0.1 to 2.0 rad/s, and the controllers respond aggressively to restore the system to nominal frequency. However, stabilization is not instantaneous due to sudden load changes, generation fluctuations, or disturbances in the system.

Several factors influence these deviations: random disturbances, especially after 10 s, can interact with system dynamics exacerbated by the variability of RERs leading to oscillatory behaviour. Limited robustness in the Neural-PI training data can also reduce the controllers’ effectiveness. Furthermore, feedback loops and event-triggered switching can amplify instability if mode transitions are delayed or coincide with disturbances.

Figure 6 presents inertia mode switching over time, showing how the base controllers adaptively adjust system inertia through the OETC mechanism. The x-axis represents time, while the y-axis indicates the selected inertia mode: low (0.00), standard (1.00), and high (2.00). Initially, from 0 to 5 s, the system remains in low-inertia mode, consistent with the pre-defined schedule. From 5 to 10 s, the system transitions to standard mode, but disturbances around 5.5 s trigger additional mode changes via the event-triggered mechanism. Beyond 10 s, the system shifts to high-inertia mode, yet frequent disturbances and active frequency tracking cause rapid oscillatory switching, reflecting the high responsiveness of the event-triggered scheme to maintain frequency stability. Table 9 presents the inertia mode switching over time.

**Table 9:** Inertia mode switching over time

Inertia mode	Time (s)
0.00 0.00	0.00 2.50
0.00	5.00
1.00	7.50
1.00	10.0
2.00	12.5
2.00	15.0
2.00	17.5
2.00	20.0

The simulation of a traditional PID controller was conducted in Python to compare its performance with the proposed OETC framework using Neural-PI controllers. The PID controller, a widely used feedback algorithm, generates outputs based on the proportional, integral, and derivative terms of the error between a setpoint and process variable. The proportional term responds to current errors, the integral term corrects accumulated past errors, and the derivative term predicts future errors. The PID simulation, implemented in the *testpid* function, dynamically updates the control output and feedback values over time, while a windup guard prevents excessive accumulation of integral error.

The simulation begins with a setpoint of zero, which later steps to one, allowing observation of the controller’s ability to drive the system to the desired value. Results are visualized in Fig. 7, with the blue curve representing PID response, the orange curve showing the Neural-PI controller response, and the black line indicating the setpoint. Initially, under low inertia, the PID controller reacts quickly, minimizing frequency deviations and stabilizing the process variable efficiently. As inertia increases to medium and high states, the response becomes slower and more sluggish, requiring longer time to reach the setpoint. The stair-step plot of inertia illustrates how inertia switching affects the system’s dynamics and the PID controller’s performance under varying conditions. Overall, while the PID maintains stability and eventually reaches the setpoint, its performance deteriorates with increasing inertia, highlighting the advantage of the adaptive Neural-PI controller in handling variable system dynamics.

### Discusion of result

The primary objective of this study was to evaluate the effectiveness of a hybrid Neural-PI controller with event-triggered switching for LFC in microgrids with variable inertia. The approach integrates machine learning principles, allowing the controller to adapt its parameters in response to dynamic system states, while reducing computational and communication overheads. Simulations were conducted in Python using Jupyter Notebook and TensorFlow to model the power system dynamics and assess performance under varying control strategies. The study specifically addressed challenges posed by increasing penetration of RERs, which reduce system inertia and compromise the effectiveness of traditional controllers such as PID and PI.

The Neural-PI event-triggered control framework demonstrated adaptive capabilities, improving system stability by applying control actions only when necessary. This mechanism efficiently balances responsiveness and resource utilization while learning from prior observations to refine control actions in real-time. Simulation results show that during the initial 15 s, the system remained stable with frequency deviations near zero. However, under high-inertia operating conditions and rapid disturbances, deviations increased sharply, revealing limitations of excessive or overly sensitive event-triggered switching. Analysis indicated that random disturbances and frequent mode transitions could induce oscillatory behavior, highlighting the need for further optimisation of switching thresholds and damping mechanisms.

Comparative results with a traditional PID controller demonstrate the superior performance of the Neural-PI controller. Figures 5, 6 and 7 illustrate that while PID response suffers from slower adaptation, higher overshoot, and delayed inertia switching, the Neural-PI controller adjusts inertia more rapidly and frequently, effectively damping disturbances and stabilizing the system. The adaptive, learning-based approach enables the Neural-PI controller to respond intelligently to changing system conditions, offering smoother frequency regulation and improved overall stability. The results also indicate that dense switching events reflect the controller’s active and adaptive management of variable inertia, emphasizing the sensitivity of system dynamics to disturbances. Table 10 presents quantitative comparison between the proposed method and the traditional method.

In summary, the proposed Neural-PI controller with online event-triggered switching provides a robust, adaptive, and efficient solution for frequency control in low-inertia, renewable-heavy microgrids. While challenges remain such as excessive switching under certain conditions and the need for comprehensive training data encompassing all nonlinear system behaviours the study confirms the potential of machine learning-based control to outperform traditional PID methods. Recommendations for further refinement include optimised threshold design, enhanced disturbance rejection strategies, and more standardized inertia modelling to improve adaptive performance.

**Table 10:** Performance comparison table

S/*N*	Metric	Proposed System	Traditional PID	Traditional PI	Improvement versus PID
1.	Maximum Frequency Deviation (rad/s)	0.05 (stable region)	0.12	0.15	58% reduction
2.	Settling Time (s)	8	12	15	33% faster
3.	Control Actions (every minute)	5	100	100	95% reduction
4.	Overshoot (%)	5%	20%	25%	75% reduction
5.	Robustness to Disturbances	High (adaptive thresholds)	Low (fixed gains)	Low	> 50% better
6.	Computational Overhead	Low (event-triggered)	High	High	90% lower

## Conclusion

The study addressed challenges in load frequency control (LFC) for islanded microgrids with high renewable energy penetration and variable system inertia. The main achievement was the design and validation of an online event-triggered LFC framework tailored to the unique dynamics of islanded microgrids. By integrating a Neural-PI controller with an event-triggered switching mechanism, the proposed approach demonstrated improved efficiency, adaptability, and robustness in frequency regulation compared to traditional PID and PI controllers.

Quantitative results highlight the advantages of the proposed framework: it achieved a 58% reduction in frequency deviation (0.05 rad/s) compared to 0.12 rad/s and 0.15 rad/s for PID and PI controllers, respectively. Control actions were significantly minimized, with only 5 activations per minute versus 100 per minute for conventional controllers, leading to a 95% reduction in unnecessary control effort. The system also exhibited faster response, with a settling time of 8 s, compared to 12 s for PID and 15 s for PI controllers. Computational overhead was reduced by 90%, enhancing microgrid reliability, reducing operational costs, and promoting sustainable energy management. Overall, the Neural-PI controller demonstrated superior system stability and responsiveness under variable inertia and high renewable penetration.

Simulations also underscored the benefits of the event-triggered control strategy, where actions are initiated only when necessary. This adaptive approach allows the Neural-PI controller to optimise control responses based on real-time disturbances and load variations. Nevertheless, challenges such as sustained oscillations arising from frequent switching and disturbances indicate the need for further refinement of the controller and the event-triggering mechanism to achieve robust practical performance.

In conclusion, the proposed Neural-PI event-triggered LFC framework demonstrates significant improvements in stability, efficiency, and adaptability over traditional controllers. With further optimisation and practical implementation, it offers a promising solution for enhancing reliability and integrating renewable energy into islanded microgrids. However, limitations were observed under back-to-back disturbances and in scenarios with unmodeled nonlinearities or insufficient training data, highlighting the need for improved robustness to ensure stability across all operating conditions.

## Data Availability

Data sets generated during the current study are available from the corresponding author on reasonable request.
